# A Molecular Study on Drug Delivery System Based on Carbon Nanotube Compared to Silicon Carbide Nanotube for Encapsulation of Platinum-Based Anticancer Drug

**DOI:** 10.15171/apb.2018.020

**Published:** 2018-03-18

**Authors:** Zahra Khatti, Seyed Majid Hashemianzadeh, Seyed Ali Shafiei

**Affiliations:** ^1^Department of Chemistry, Iran University of Science and Technology, Tehran, Iran.; ^2^Molecular Simulation Research Laboratory, Department of Chemistry, Iran University of Science and Technology, Tehran, Iran.; ^3^Neurology and Neuroscience Research Center, Qom University of Medical Sciences, Qom, Iran.

**Keywords:** Molecular Dynamic Simulation, Carbon nanotube, Silicon Carbide nanotube, Platinum-based anticancer drug

## Abstract

***Purpose:*** Drug delivery has a critical role in the treatment of cancer, in particular, carbon nanotubes for their potential use in various biomedical devices and therapies. From many other materials which could be more biocompatible and biodegradable and which could form single-walled nanotubes, silicon carbide was selected.

***Methods:*** To compare two drug delivery systems based on single-walled nanotubes, molecular dynamic simulations were applied and encapsulation behavior of the drug carboplatin was investigated inside the silicon carbide nanotube and the carbon nanotube.

***Results:*** Localization of the carboplatin inside the nanotubes indicated that the carboplatin moves throughout the tubes and possesses a greater probability of finding the drug molecule along the nanotubes in the first quarter of the tubes. The energy analysis exhibited the lowest free energy of binding belongs to the encapsulation of the drug carboplatin in the silicon carbide nanotube, about -145 Kcal/mol.

***Conclusion:*** The results confirmed that the silicon carbide nanotube is a more suitable model than the carbon nanotube for drug delivery system based on nanotubes as a carrier of platinum-based anticancer drugs.

## Introduction

Carbon nanotubes (CNTs), due to their unique atomic configuration, mechanical, optical and electronic properties, have been envisioned in designing biomedical devices and therapies on novel delivery platforms.^1-5^ The physicochemical versatility of carbon nanotubes is related to their high surface-area-to-volume ratios and facile functionalization along the nanotube axis and a great inner content that can be filled with the desired drug molecules.^6-8^ Additionally, the ability of single-walled carbon nanotubes (SWCNT) to incorporate inside cells has been proven, independent of cell type and functional groups linked to the nanotubes.^9,10^ In vivo assays have demonstrated that SWCNTs as carriers have no obvious toxicity,^11^ and in animal models have demonstrated the efficacy of drug-loaded CNTs through targeting tumors.^6,12^ Whereas water is the main component in biological systems and CNTs are hydrophobic, heterogeneous CNTs could be considered including silicon carbide nanotube (SiCNT) in aqueous media.^13^ In previous studies, several advantages have been shown for SiCNTs compared to CNTs. First, there is the relative stability increase from the CNTs to SiCNTs when the ratio of Si over C is 50:50, due to alternative sp2 and sp3 hybridization bond structures that are more stable than a smooth-walled tube.^14^ Additionally, the external surface of SiCNTs has higher reactivity than that of CNTs to facilitate aimed side wall functionalization.^15,16^ Furthermore, the experimental results prove the biocompatibility of silicon nanotubes and hence an alternative option for applications in nanomedicine.^17,18^ Platinum-based anticancer drugs are used to treat many types of solid tumors via binding to DNA and inducing cellular apoptosis, despite their adverse side effects,^19,20^ so that many of these side effects for healthy cells can be greatly reduced by nanoscale drug delivery. A fast and reliable tool to evaluate theoretically such systems is molecular modeling, which could interpret the details of the interaction between the drug and both the DNA and the nanostructures.^21^ On the other hand,‏ our previous study allowed the assessment of a drug delivery system caused by another nanotube apart from CNTs, as carrier on theoretical level for the first time.^22^ Therefore, in the present study, molecular dynamics (MD) simulation was employed to investigate another so-named nanotube SiCNT as delivery system compared to CNT. Carboplatin (diammineplatinum(II) cyclobutane-1,1-dicarboxylate) was selected as a drug model because it encompasses fewer adverse effects and has greater water solubility than other platinum agents.^23-25^ Our simulations have been performed to assess the encapsulation behavior and localization of the anticancer drug carboplatin inside pristine CNT and SiCNT. For this purpose, the placement of the drug inside nanotubes and the free energy calculations were implemented to determine the preference between the two predicted drug delivery systems.

## Materials and Methods

### Systems preparation

In the present work, the zigzag open-ended single-walled nanotubes with 14 Å diameter and 40 Å length were considered, so (18, 0) carbon nanotube and (14, 0) silicon carbide nanotube were applied. Molecular dynamic simulations were applied for two systems; both of them containing a carboplatin molecule at a position along the CNT and SiCNT z-axis, i.e., drug_CNT and drug_SiCNT. Two systems were solvated in an aqueous solution. The parameters of CNT were modeled using the AMBER99SB force field.^26,27^ All the Lennard-Jones parameters and partial charge values for the carbon and silicon atoms for SiCNT were obtained from density functional theory calculations, summarized in Table 1.^14,28^ Other parameters were taken from the DREIDING force field and used in MD simulations. The structure of carboplatin was obtained from our previous works,^22,29^ and the parameters were identified from the literature and the GAFF;^30,31^ additionally, the carboplatins’ partial charges using the RESP module of AMBER 12 were calculated. The desired structures were immersed in a periodic water box of octagonal shape with a minimal distance of 12 Å from the system surface. Two systems were solvated with a TIP3P solvation model.^32^

**Table 1:** Lennard-Jones parameters and partial charges for SiCNT atoms

**Atom**	**ε (kcal/mol)**	**σ (Å)**	**Atomic Charge (e)**
C	0.086	3.4	0.45
Si	0.469	3.7364	-0.45

### Molecular dynamics simulations

Molecular dynamics simulations were performed by employing the AMBER 12 simulation package.^33^ All simulations were conducted under the isothermal-isobaric ensemble at 1 atm and 300 K using the SANDER module in the AMBER 12 program. Constant temperature and pressure were maintained using Langevin dynamics.^34^ Bond lengths containing hydrogen atoms were constrained using the SHAKE algorithm.^35^ Periodic boundary conditions were applied and the long-range electrostatic interactions were handled with the particle mesh Ewald method; the nonbonded interactions were treated with cutoff at 12Å.^36^ Each simulation in energy minimization stage included 5000 steps for solvent and 5000 steps for solute relaxation. All systems were then heated from 0K to 300K for 120 ps and equilibrated at 300K for 200 ps. The production stages were run for 10 ns with a time step of 2 fs, and the trajectories for structural coordinates were saved every 1 ps for data analysis. Configuration analysis of the trajectories was performed with the ptraj module included in AmberTools14. Additionally, binding free energies were evaluated using the MM/PBSA (molecular mechanics/Poisson-Boltzmann surface area) method by previous experiment.^29,37^ The analysis of the interaction energies between the nanotubes and the drug was accomplished with AmberTools14 and implementation of MMPBSA.py script.

## Results

**Figure 1:**
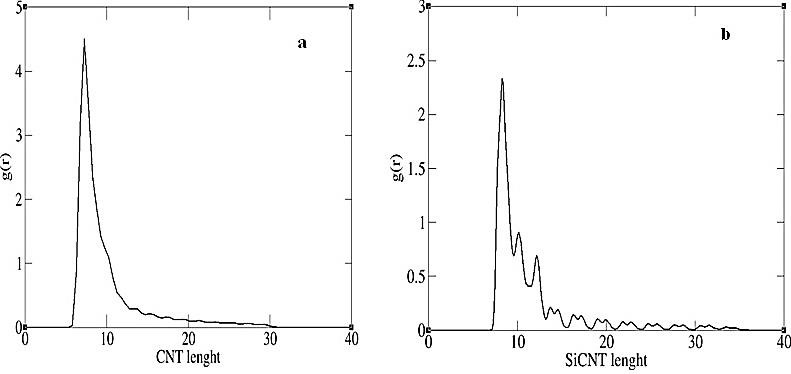
The RDF plot between the carboplatin center of mass and (a) the carbon atoms of the CNT, (b) the carbon and silicon atoms of the SiCNT

**Figure 2:**
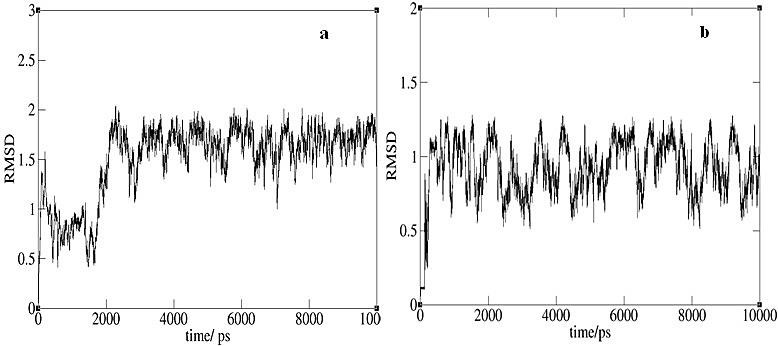
RMSD plots of encapsulation of the carboplatin inside the (a) CNT, (b) SiCNT.

The results from MD simulations of the two systems revealed that throughout the simulation time, both encapsulated carboplatin molecules resided inside the CNT and SiCNT cavity. Time-averaged radial distribution functions (RDF) were calculated to assess the localization of carboplatin inside the nanotubes (Figure 1), also RMSD plots were illustrated the drug movement inside the carbon and silicon carbide nanotubes (Figure 2). Moreover, MM/PBSA analysis of the trajectories was performed to estimate the binding free energy of the drug_CNT and the drug_SiCNT. Binding and absolute free energies of molecules are evaluated using this attractive method:

$$\Delta G_{binding}=\left[G_{complex}\right]-\left[G_{drug}\right]-\left[G_{CNT}\right]$$

Additionally, separation of the total free energy of binding into the van der Waals, electrostatic, and solute-solvent interactions, and discussion of each of the terms can be allowed and is calculated from:

$$\Delta G_{tot}=\Delta E_{MM}+\Delta G_{solv}-T\Delta S$$

$$\Delta E_{MM}=E_{int}+E_{vdW}+E_{ele}$$

$$\Delta G_{solv}^{PBSA}=\Delta G_{solv}^{nonpolar}+\Delta G_{solv}^{electrostatic}$$

where the molecular mechanics energy of the molecule (∆E_MM_) includes the van der Waals (E_vdW_), internal energy (bonds, angles and dihedrals) (E_int_) and the electrostatic energy (E_ele_) terms. The solvation energy (G_solv_) containing the polar and nonpolar parts is obtained from an implicit solvent description. Table 2 summarizes all energy terms for the two simulated systems in order to evaluate binding free energy using the MM/PBSA method.

## Discussion

### Localization of carboplatin inside the CNT and SiCNT

To assess the localization of carboplatin inside the nanotubes, time-averaged radial distribution functions (RDF) were calculated. Figure 1a illustrates the RDF plot between the carboplatin center of mass and the carbon atoms of the CNT. In addition, Figure 1b illustrates the RDF plot between the carboplatin center of mass and the carbon and silicon atoms of the SiCNT. Both plots indicate that the drug moves throughout the tubes and there is a greater probability of finding the drug along the CNT and SiCNT in the first quarter of the tubes. Since drug exposure at the end of the nanotube is an important option for the drug molecules to release,^38^ the encapsulation and the preferential position of the carboplatin inside the tubes have a significant contribution in drug delivery.

### Energy analysis

With regard to RMSD plots of drug movement inside the carbon and silicon carbide nanotubes (Figure 2), it is obvious that the two systems have stable equilibration, so it is possible to assess the thermodynamic properties of systems by sampling in simulation time and achieving the localization and the encapsulation behavior of the carboplatin inside the CNT and SiCNT. From the results of energy terms in Table 2, the total binding free energy in the drug_CNT system with an average of about −46 kcal mol^−1^ and the drug_SiCNT with −145 kcal mol^−1^ remains constant. It is derived that the dominant contribution of interactions is related to nonbonded van der Waals energy; evidently indicating that encapsulation of carboplatin inside the silicon carbide nanotube has stronger vdW interactions. The solvation free energies (GsolvPBSA) have a positive and‏ small contribution of the binding free energy for both systems (Table 2), due to the hydrophobic structures of nanotubes; however, this is slightly reduced in the drug_SiCNT system. Whereas the partial charge on carbon atoms of CNT is zero, this reduction of solvation free energies can be related to partial charges on carbon and silicon atoms of the silicon carbide nanotube’s structure. Regard these results, the larger van der Waals value belongs to the drug_SiCNT, so the carboplatin drug has stronger interaction inside the SiCNT than it does inside the carbon nanotube. Comparison of these energy profiles reveals that the carboplatin molecule prefers to spend more time inside the SiCNT until it reaches the target cell. Consequently, using SiCNT nanotube as a platinum drug carrier can be the more suitable option for drug delivery with this type of nanostructure.

**Table 2:** Energy values for encapsulation of drug inside the nanotubes

Complex	drug_CNT	drug_SiCNT
E_vdW_	-50.7566	-147.1443
E_ele_	-0.9297	-2.9051
G_solv_^ele^	7.0830	6.0232
G_solv_^nonpolar^	-1.7366	-1.0369
E_MM_	-51.6863	-150.0495
G_solv_^PBSA^	5.3464	4.9863
∆G_binding_	-46.3399	-145.0631

All values in this table are in kcal/mol.

## Conclusion

Molecular dynamic simulations to investigate the encapsulation behavior of the drug carboplatin inside the silicon carbide nanotube were applied as a comparison with the carbon nanotube as an anticancer drug delivery system based on single-walled nanotubes. The RDF plots show the localization of carboplatin inside the nanotubes, indicating that the drug moves throughout the tubes and has a greater probability of finding the carboplatin along the CNT and SiCNT in the first quarter of the tubes. Additionally, the binding free energy profiles in the encapsulation of drug inside both systems were investigated. The results confirmed that the appropriate drug delivery system for platinum drug is the use of SiCNTs to CNTs. Since the free energy of binding in the silicon carbide nanotube is about three times that of the carbon nanotube, the length of time remaining for the drug in this system will be greater, and the probability of releasing the drug will be less than with carbon nanotube, before reaching the target cells.

## Acknowledgments

The authors give special thanks to S. Skies for editing this manuscript.

## Ethical Issues

Not applicable.

## Conflict of Interest

The authors declare no conflict of interests.
